# Lesão Renal Independente do Volume: Por que a Nefropatia Induzida por Contraste pode Ocorrer Mesmo com Doses Ultrabaixas de Contraste

**DOI:** 10.36660/abc.20260016

**Published:** 2026-04-23

**Authors:** Fahri Çakan

**Affiliations:** 1 Alanya Alaaddin Keykubat University Antalya Turquia Alanya Alaaddin Keykubat University – Cardiology, Antalya – Turquia

**Keywords:** Meios de Contraste, Nefropatias, Intervenção Coronária Percutânea, Injúria Renal Aguda

**Prezado Editor,**

Analisei com grande interesse o artigo intitulado "Impacto do volume de contraste utilizado após procedimentos coronários percutâneos em pacientes com risco de nefropatia induzida por contraste", de autoria de Freitas et al.^1^ Esta análise de subgrupo post-hoc, concebida para avaliar a gestão do risco de nefropatia induzida por contraste (NIC), fornece dados clinicamente valiosos sobre a interação entre o tipo e o volume de contraste, particularmente em populações de pacientes de alto risco (n=2.268).^1^

A NIC é uma das complicações que nós, cardiologistas intervencionistas, antecipamos e sempre temos em mente antes de iniciar um procedimento. Aliás, essa preocupação é maior do que a expectativa de complicações técnicas que possam ocorrer durante o procedimento. Outro ponto importante é que, em alguns pacientes, mesmo com o uso de uma quantidade significativa de contraste, não há alteração nos valores renais. A maioria dos cardiologistas já se deparou com essa situação. Nesse ponto, fatores como diabetes, idade avançada e doença renal pré-existente podem influenciar o resultado, mesmo com volumes baixos de contraste, por meio da "modulação do risco individual".^2^

Dividir os grupos em intervalos de 150 ml apresenta alguns problemas e oferece uma solução. Primeiro, a distribuição dos participantes entre os grupos é bastante diferente. Dado o grande número de participantes, mesmo a menor diferença poderia ser significativa. No entanto, curiosamente, devido ao resultado negativo do estudo, a diferença no número de participantes entre os grupos atenua essa situação. Portanto, acredito que um volume de corte menor deva ser usado para a análise conjunta, tanto para equilibrar a proporção de participantes entre os grupos quanto para reduzir a tendência a um erro do tipo 2.

Em procedimentos coronários, é bastante difícil estimar com exatidão a quantidade de contraste efetivamente utilizada na prática clínica. Isso ocorre porque uma quantidade considerável de contraste radiopaco é expelida pela linha de injeção, pelo cateter, pelo indeflador e durante a lavagem da linha. Considerando que nem todo esse contraste entra no corpo do paciente, a quantidade efetivamente utilizada é menor. Esse volume pode variar de aproximadamente 25 a 50 ml, podendo ser ainda maior em procedimentos complexos e em pacientes submetidos a cirurgia de revascularização miocárdica. Neste artigo, o volume médio de contraste para o Grupo I é de 75,3 ± 28,0 ml. Para o Grupo II, é de 188,6 ± 46,9 ml. Pode-se afirmar que pelo menos 20 a 25 ml desses valores não chegam a entrar no leito vascular do paciente, sendo utilizados apenas para o funcionamento do sistema. Considerando esses volumes perdidos em ambos os grupos, podemos supor que o Grupo II utiliza quase três vezes mais contraste efetivo.

O desenvolvimento de NIC com o uso de alto contraste já é conhecido. Os autores observaram, nas limitações, que o número de pacientes que utilizaram alto contraste foi relativamente pequeno. No entanto, a comparação entre grupos com volumes baixos (<200-300 cc) é importante para a prática clínica, pois o poder estatístico foi mantido apesar de todas as restrições e análises. Embora os fatores de risco clássicos e seus níveis de efeito para o desenvolvimento de NIC estejam bem definidos, se sabe que a NIC pode se desenvolver mesmo com volumes muito baixos. Os rins são vulneráveis ao contraste em alto volume.^3^ Contudo, isso destaca um ponto importante nos resultados deste estudo. Apesar de uma diferença de contraste de quase três vezes e do uso de contraste de baixa osmolaridade, a ausência de diferença significativa entre os dois grupos sugere que, se um paciente vai desenvolver NIC, ele certamente a desenvolverá; a importância do volume e da osmolaridade média é negligenciável. Nesse ponto, se pode considerar que outros fatores na fisiopatologia da NIC podem ser secretamente mais eficazes. Isso explica por que a fisiopatologia da NIC não depende exclusivamente do volume e por que processos como isquemia medular renal e toxicidade tubular podem ser desencadeados mesmo em doses muito baixas em pacientes de alto risco.^4^ A incidência de 14,8% de NIC observada no grupo de baixo volume (Grupo I) corrobora a teoria de lesão renal independente do volume em pacientes de alto risco relatada na literatura.^4,5^ Se a NIC se desenvolver em pacientes mesmo com baixos volumes de contraste, será essencial tomar medidas de proteção para todos os pacientes, sem exceção. Por essa razão, acredito que seja hora de deixarmos de lado a importância do tipo e do volume de contraste para o desenvolvimento da NIC.
