# Supplementary material for: Lesão Renal Independente do Volume: Por que a Nefropatia Induzida por Contraste pode Ocorrer Mesmo com Doses Ultrabaixas de Contraste
Source: Arq Bras Cardiol. 2026 Apr 23;123(4):e20260016. [Article in Portuguese] doi: 10.36660/abc.20260016 (PMC13155796; doi:10.36660/abc.20260016)
Supplement: Translation

# Translation: Volume-Independent Renal Injury: Why Contrast-Induced Nephropathy Can Occur Even at Ultra-Low Contrast Doses

**DOI:** 10.36660/abc.20260016i

**Authors:** Fahri Çakan

**Affiliations:** 1 Antalya Türkiye Alanya Alaaddin Keykubat University – Cardiology, Antalya – Türkiye

**Keywords:** Contrast Media, Kidney Diseases, Percutaneous Coronary Intervention, Acute Kidney Injury

**Dear Editor,**

I reviewed with great interest the article titled "Impact of Contrast Volume Used After Percutaneous Coronary Procedures in Patients at Risk for Contrast-Induced Nephropathy," authored by Freitas et al.^1^ This post-hoc subgroup analysis, designed to assess contrast-induced nephropathy (CIN) risk management, provides clinically valuable data on the interaction between contrast type and volume, particularly in high-risk patient populations (n=2,268).^1^

CIN is one of the complications that we, as invasive cardiologists, anticipate and always keep in mind before starting a case. In fact, this feeling is greater than the expectation of technical complications that may occur during the procedure. Another point is that in some patients, even when a significant amount of contrast is used, there is no change in kidney values. Most cardiologists have experienced this situation. At this point, factors such as diabetes, advanced age, and existing kidney disease can cause the outcome to change even at low volumes by "modulating individual risk."^2^

Dividing the groups into 150 ml actually brings certain problems and a solution with it. First of all, the distribution of participants in the groups is quite different. Considering that the number of participants is also high, even the slightest difference can be expected to be significant. However, the interesting point is that, due to the negative outcome of the study, the difference in participants between groups suppresses this situation. Therefore, I believe that a lower volume cutoff should be applied for grouping, both to balance the ratio of participants between groups and to reduce the tendency for type 2 error.

In coronary procedures, it is quite difficult to estimate exactly how much contrast agent is actually used in real life. This is because a considerable amount of radiopaque agent is expelled within the injection line, within the catheter, within the indeflator, and during line flushing. Assuming that all of this does not enter the patient's body, the amount of contrast agent actively used is less. This volume can range from approximately 25-50 ml, and may be even higher in complex procedures and bypass patients. In this article, the average contrast volume for Group I is stated as 75.3 ± 28.0 mL. For Group II, it is 188.6 ± 46.9 mL. It can be said that at least 20-25 mL of these figures does not actually enter the patient's vascular bed but is only used for the system to function. Considering these lost volumes for both groups, we can assume that nearly three times as much effective contrast is used in Group 2.

The development of CIN with high contrast use is already known. The authors noted in the limitations that the number of patients using high contrast was relatively small. However, comparing groups with low volumes (<200-300 cc) is important for clinical practice. This is because statistical power was maintained despite all the constraints and analyses. Although the classic risk factors and their levels of effect for CIN development are well defined, it is known that CIN can develop even at very low volumes. The kidneys are vulnerable to high-volume contrast.^3^ However, it highlights an important point in the results of this study. Despite a contrast difference of nearly threefold and despite the use of low-osmolar contrast, the absence of a significant difference between the two groups suggests that if a patient is going to develop CIN, he will; the importance of volume and media osmolarity is negligible. At this point, it can be considered that other players in the pathophysiology of CIN may be secretly more effective. It explains that the pathophysiology of CIN is not solely volume-dependent, and that processes such as renal medullary ischemia and tubular toxicity can be triggered even at very low doses in high-risk patients.^4^ The 14.8% incidence of CIN observed in the low-volume group (Group I) supports the theory of volume-independent renal injury in high-risk patients reported in the literature.^4,5^ If CIN develops in patients even at low volumes, it will be essential to take protective measures for every patient without question. For this reason, I believe it is time to set aside the importance of contrast type and volume for CIN development.
