# Supplementary material for: Lesão Renal Independente do Volume: Por que a Nefropatia Induzida por Contraste pode Ocorrer Mesmo com Doses Ultrabaixas de Contraste
Source: Arq Bras Cardiol. 2026 Apr 23;123(4):e20260016. [Article in Portuguese] doi: 10.36660/abc.20260016 (PMC13155796; doi:10.36660/abc.20260016)
Supplement: Related texts

# Reply: Carta-resposta

**Authors:** Rafaela Andrade Penalva Freitas, José de Ribamar Costa

**Affiliations:** 1 Instituto Dante Pazzanese de Cardiologia São Paulo SP Brasil Instituto Dante Pazzanese de Cardiologia, São Paulo, SP – Brasil; 2 Hospital São Domingos São Luís MA Brasil Hospital São Domingos, São Luís, MA – Brasil

Agradeço sinceramente pelo interesse demonstrado no nosso artigo e pela leitura atenta e reflexiva do manuscrito. Cartas como a sua enriquecem o debate científico e nos ajudam a refinar a interpretação dos dados, especialmente em um tema tão relevante e ainda controverso como a nefropatia induzida por contraste (NIC).

Concordo plenamente com sua observação de que a NIC permanece uma preocupação constante entre os cardiologistas. É fato bem conhecido que, em pacientes de alto risco (diabetes, idade avançada, doença renal pré-existente), o limiar para o desenvolvimento de lesão renal pode ser consideravelmente mais baixo, e fatores individuais modulam fortemente o risco, como bem destacou ao citar a modulação individual do risco.

Na população em geral, a incidência de NIC é inferior a 2%.^1^ Em estudo publicado em 2020, que avaliou 82.683 pacientes submetidos à intervenção coronária percutânea, a incidência observada foi de 5,73%, valor semelhante ao relatado pelo Registro NCDR CathPCI, divulgado em 2018.^2^ Ressalta-se, contudo, que em pacientes classificados como de alto risco para o desenvolvimento dessa complicação, as taxas podem ultrapassar 30%, a depender da quantidade de fatores de risco presentes individualmente.^1,3,4^

O estudo IDPC foi um estudo clínico randomizado, unicêntrico, que comparou contrastes iodados de baixa osmolaridade (ioxaglato) e iso-osmolaridade (iodixanol) na prevenção de nefropatia induzida por contraste (NIC) em pacientes de alto risco submetidos a procedimentos coronários percutâneos diagnósticos e/ou terapêuticos. Foram incluídos 2.268 pacientes com pelo menos um fator de alto risco (idade >70 anos, diabetes, doença renal crônica não dialítica, insuficiência cardíaca, choque cardiogênico ou síndrome coronariana aguda). O desfecho primário foi a ocorrência de NIC, definida como aumento relativo >25% e/ou absoluto >0,5 mg/dL da creatinina entre o 2° e o 5° dia após a exposição ao contraste. A incidência global de NIC foi de 15%, sem diferença significativa entre contraste iso-osmolar (15,2%) e baixa osmolaridade (15,1%; P>0,99), inclusive nas análises de subgrupos. Também não houve diferença quanto à necessidade de diálise em 30 dias ou mortalidade. Concluiu-se que, em pacientes de alto risco, a ocorrência de NIC foi semelhante independentemente do tipo de contraste utilizado.^5^

Em nossa instituição, como prática rotineira, exames diagnósticos e procedimentos coronários sempre foram realizados com a menor quantidade possível de contraste. A média de constrate utilizada no estudo IDPC foi 89,5mL.^5^ Para a análise dos grupos, foi aplicada regressão logística com o objetivo de avaliar a ocorrência de desfechos e sua relação com a função renal. A partir da análise estatística, se identificou que o ponto de corte de 150 mL de contraste se mostrou o mais adequado para essa avaliação.

Sabemos que a adoção de um ponto de corte inferior a 150 mL para a estratificação dos grupos, resultou em grupos desbalanceados em tamanho, mas vale lembrar que as populações são muito semelhantes nas suas características. De fato, em análises post-hoc como a nossa, o equilíbrio entre os grupos nem sempre é ideal, e um corte mais baixo (por exemplo, próximo de 100–120 mL, ou ajustado por peso corporal/creatinina) poderia trazer maior granularidade e talvez maior poder estatístico para detectar diferenças sutis em volumes muito baixos. Trata-se de uma limitação inerente ao desenho e sua crítica é pertinente; ela reforça a necessidade de estudos prospectivos com estratificações pré-definidas mais refinadas.

Quanto à questão do volume efetivo de contraste que chega ao paciente, sua observação é extremamente válida e frequentemente subestimada na literatura. Os 20–50 mL (ou mais em procedimentos complexos) que permanecem na linha de injeção, no cateter, realmente não contribuem para a exposição sistêmica. Esse ponto fortalece a discussão sobre a dificuldade de estimar o volume real na prática clínica e reforça a importância de técnicas de minimização de contraste desperdiçado (sistemas de injeção automatizados, cateteres de baixo volume, etc).

No serviço de cardiologia invasiva do IDPC, integra a rotina da sala de hemodinâmica, a aspiração do contraste residual presente no cateter e na linha injetora ao término do procedimento, com sua devolução ao frasco original e posterior contabilização do volume total administrado ao final do exame.

O achado mais intrigante do nosso trabalho^6^ — e o que motivou o título da sua carta — foi precisamente a ausência de diferença significativa na incidência de NIC entre os grupos, apesar da diferença substancial de volume e do uso de contraste de baixa osmolalidade em ambos. A incidência de ~14,8% no grupo de baixo volume é, de fato, importante e se alinha com relatos na literatura de lesão renal em pacientes de altíssimo risco mesmo com volumes muito baixos (<50–100 mL). Isso apoia a noção de que, em certos perfis de pacientes, a NIC pode ter um componente volume-independente (ou pouco dependente de volume) significativo, mediado por mecanismos como isquemia medular grave, toxicidade tubular direta em nefrons já vulneráveis, disfunção endotelial, estresse oxidativo e inflamação — processos que podem ser iniciados por doses mínimas em indivíduos predispostos.^7^

No entanto, é importante não generalizar excessivamente essa conclusão. A literatura ainda demonstra uma relação dose-resposta clara na maioria dos cenários:^8,9^ volumes >100–140 mL (ou >3–4 mL/kg ou V/CrCl >3,7) aumentam consistemente o risco em análises multivariadas, especialmente em populações mistas. O que seu comentário destaca — e com razão — é que, no extremo alto risco, o volume deixa de ser o principal modulador, e outros fatores (hipoperfusão, anemia, hipotensão peri-procedimento, microembolização ateromatosa, entre outros) podem dominar a fisiopatologia. Nesse subgrupo, a NIC pode ser mais *"contrast-associated"* do que estritamente *"contrast-induced"*, e medidas de proteção (hidratação adequada, retirada de nefrotóxicos, uso de estatina de alta intensidade, minimização rigorosa de contraste) devem ser aplicadas universalmente, independentemente do volume planejado.

Em resumo, essa discussão traz uma reflexão profunda e necessária sobre o tema, considerando uma visão abrangente do papel da escolha do meio de contraste, vulnerabilidade individual e os mecanismos não volumétricos. Concordo que é momento de repensar estratégias preventivas como universais e não apenas volume-dependentes.

Agradeço novamente pela contribuição valiosa ao debate.

## References

[1] Aspelin P, Aubry P, Fransson SG, Strasser R, Willenbrock R, Berg KJ, et al. Nephrotoxic Effects in High-Risk Patients Undergoing Angiography. N Engl J Med. 2003;348(6):491-9. doi: 10.1056/NEJMoa021833.10.1056/NEJMoa02183312571256

[2] Amin AP, McNeely C, Spertus JA, Bach RG, Frogge N, Lindner S, et al. Incremental Cost of Acute Kidney Injury after Percutaneous Coronary Intervention in the United States. Am J Cardiol. 2020;125(1):29-33. doi: 10.1016/j.amjcard.2019.09.042.10.1016/j.amjcard.2019.09.04231711633

[3] Mehran R, Aymong ED, Nikolsky E, Lasic Z, Iakovou I, Fahy M, et al. A Simple Risk Score for Prediction of Contrast-İnduced Nephropathy after Percutaneous Coronary İntervention: Development and İnitial Validation. J Am Coll Cardiol. 2004;44(7):1393-9. doi: 10.1016/j.jacc.2004.06.068.10.1016/j.jacc.2004.06.06815464318

[4] Budano C, Levis M, D’Amico M, Usmiani T, Fava A, Sbarra P, et al. Impact of Contrast-Induced Acute Kidney Injury Definition on Clinical Outcomes. Am Heart J. 2011;161(5):963-71. doi: 10.1016/j.ahj.2011.02.004.10.1016/j.ahj.2011.02.00421570530

[5] Freitas RAP, Tanajura LF, Mehran R, Chamié D, Chaves A, Centemero M, et al. Ioxaglate versus IoDixanol for the Prevention of Contrast-Induced Nephropathy: The IDPC Trial. J Invasive Cardiol. 2023;35(6):E281-90. doi: 10.25270/jic/21.00249.10.25270/jic/21.0024937220640

[6] Freitas RAP, Tanajura LFL, Sousa AGMR, Feres F, Costa JR. Impact of Contrast Volume Used after Percutaneous Coronary Procedures in Patients at Risk for Contrast-Induced Nephropathy. Arq Bras Cardiol. 2025;122(12):e20250270. doi: 10.36660/abc.20250270.10.36660/abc.20250270PMC1297836141615170

[7] Mehran R, Dangas GD, Weisbord SD. Contrast-Associated Acute Kidney Injury. N Engl J Med. 2019;380(22):2146-55. doi: 10.1056/NEJMra1805256.10.1056/NEJMra180525631141635

[8] Nie Z, Liu Y, Wang C, Sun G, Chen G, Lu Z. Safe Limits of Contrast Media for Contrast-Induced Nephropathy: A Multicenter Prospective Cohort Study. Front Med. 2021;8:701062. doi: 10.3389/fmed.2021.701062.10.3389/fmed.2021.701062PMC841779434490295

[9] Liu Y, Chen SQ, Duan CY, Tan N, Chen JY, Zhou YL, et al. Contrast Volume-to-Creatinine Clearance Ratio Predicts the Risk of Contrast-Induced Nephropathy after Percutaneous Coronary Intervention in Patients with Reduced Ejection Fraction. Angiology. 2015;66(7):625-30. doi: 10.1177/0003319714548442.10.1177/000331971454844225158831

# Reply

**Authors:** Rafaela Andrade Penalva Freitas, José de Ribamar Costa

**Affiliations:** 1 São Paulo SP Brazil Instituto Dante Pazzanese de Cardiologia, São Paulo, SP – Brazil; 2 São Luís MA Brazil Hospital São Domingos, São Luís, MA – Brazil

Thank you sincerely for your interest in our article and for your careful and thoughtful reading of the manuscript. Letters like yours enrich the scientific debate and help us refine the interpretation of the data, especially on a topic as relevant and still controversial as contrast-induced nephropathy (CIN).

I fully agree with your observation that CIN remains a constant concern among cardiologists. It is a well-known fact that, in high-risk patients (diabetes, advanced age, pre-existing kidney disease), the threshold for developing kidney damage can be considerably lower, and individual factors strongly modulate the risk, as you rightly pointed out when citing individual risk modulation.

In the general population, the incidence of CIN is less than 2%.^1^ In a study published in 2020, which evaluated 82,683 patients undergoing percutaneous coronary intervention, the observed incidence was 5.73%, a value similar to that reported by the NCDR CathPCI Registry, published in 2018.^2^ It should be noted, however, that in patients classified as high-risk for developing this complication, rates can exceed 30%, depending on the number of risk factors present individually.^1,3,4^

The IDPC study was a randomized, single-center clinical trial that compared low-osmolarity (ioxaglate) and iso-osmolarity (iodixanol) iodinated contrast agents in the prevention of contrast-induced nephropathy (CIN) in high-risk patients undergoing diagnostic and/or therapeutic percutaneous coronary procedures. 2,268 patients with at least one high-risk factor (age >70 years, diabetes, non-dialysis chronic kidney disease, heart failure, cardiogenic shock, or acute coronary syndrome) were included. The primary outcome was the occurrence of CIN, defined as a relative increase >25% and/or absolute increase >0.5 mg/dL in creatinine between the 2nd and 5th day after contrast exposure. The overall incidence of CIN was 15%, with no significant difference between iso-osmolar (15.2%) and low-osmolarity (15.1%; P>0.99) contrast agents, including in subgroup analyses. There was also no difference in the need for dialysis within 30 days or mortality. It was concluded that, in high-risk patients, the occurrence of CIN was similar regardless of the type of contrast agent used.^5^

In our institution, as routine practice, diagnostic tests and coronary procedures have always been performed with the smallest possible amount of contrast. The average contrast used in the IDPC study was 89.5 mL.^5^ For the analysis of the groups, logistic regression was applied to evaluate the occurrence of outcomes and their relationship with renal function. From the statistical analysis, it was identified that the cutoff point of 150 mL of contrast proved to be the most appropriate for this evaluation.

We know that adopting a cutoff point lower than 150 mL for group stratification resulted in unbalanced groups in size, but it is worth remembering that the populations are very similar in their characteristics. In fact, in post-hoc analyses like ours, the balance between groups is not always ideal, and a lower cutoff (for example, closer to 100–120 mL, or adjusted for body weight/creatinine) could provide greater granularity and perhaps greater statistical power to detect subtle differences in very low volumes. This is an inherent limitation of the design, and its criticism is pertinent; it reinforces the need for prospective studies with more refined pre-defined stratifications.

Regarding the issue of the effective volume of contrast that reaches the patient, your observation is extremely valid and is frequently underestimated in the literature. The 20–50 mL (or more in complex procedures) that remain in the injection line, catheter, do not actually contribute to systemic exposure. This point strengthens the discussion about the difficulty of estimating the real volume in clinical practice and reinforces the importance of techniques for minimizing wasted contrast (automated injection systems, low-volume catheters, etc.).

In the invasive cardiology service at IDPC, the routine in the hemodynamics room includes aspirating the residual contrast present in the catheter and injection line at the end of the procedure, returning it to the original vial, and subsequently accounting for the total volume administered at the end of the examination.

The most intriguing finding of our work^6^ — and what motivated the title of your letter — was precisely the absence of a significant difference in the incidence of CIN between the groups, despite the substantial difference in volume and the use of low-osmolality contrast in both. The incidence of ~14.8% in the low-volume group is, in fact, important and aligns with reports in the literature of kidney injury in very high-risk patients, even with very low volumes (<50–100 mL). This supports the notion that, in certain patient profiles, CIN may have a significant volume-independent (or slightly volume-dependent) component, mediated by mechanisms such as severe bone marrow ischemia, direct tubular toxicity in already vulnerable nephrons, endothelial dysfunction, oxidative stress, and inflammation — processes that can be initiated by minimal doses in predisposed individuals.^7^

However, it is important not to overgeneralize this conclusion. The literature still demonstrates a clear dose-response relationship in most scenarios^8,9^: volumes >100–140 mL (or >3–4 mL/kg or V/CrCl >3.7) consistently increase risk in multivariate analyses, especially in mixed populations. What your comment highlights—and rightly so—is that, at the extreme high risk, volume ceases to be the primary modulator, and other factors (hypoperfusion, anemia, peri-procedural hypotension, atherosclerotic microembolization, among others) may dominate the pathophysiology. In this subgroup, CIN may be more "contrast-associated" than strictly "contrast-induced," and protective measures (adequate hydration, withdrawal of nephrotoxic agents, use of high-intensity statins, rigorous minimization of contrast) should be universally applied, regardless of the planned volume.

In summary, this discussion brings a profound and necessary reflection on the topic, considering a comprehensive view of the role of contrast medium choice, individual vulnerability, and non-volumetric mechanisms. I agree that it is time to rethink preventive strategies as universal and not just volume-dependent.

Thank you again for your valuable contribution to the discussion.
